# Current evidence for designing self-management support for underserved populations: an integrative review using the example of diabetes

**DOI:** 10.1186/s12939-023-01976-6

**Published:** 2023-09-11

**Authors:** Ian Litchfield, Tim Barrett, Julian Hamilton-Shield, Theresa Moore, Parth Narendran, Sabi Redwood, Aidan Searle, Suma Uday, Jess Wheeler, Sheila Greenfield

**Affiliations:** 1 Institute of Applied Health Research, University of Birmingham, Birmingham, B15 2TT UK; 2 Institute of Cancer and Genomic Sciences, University of Birmingham, Birmingham, B15 2TT UK; 3 Diabetes and Endocrinology, Birmingham Women’s and Children’s Hospital, Birmingham, B4 6NH UK; 4 Translational Health Sciences, Bristol Medical School, University of Bristol, Bristol, BS1 2NT UK; 5 The Royal Hospital for Children in Bristol, Bristol, BS2 8BJ UK; 6 NIHR Bristol BRC Nutrition Theme, University Hospitals Bristol and Weston Foundation Trust, Bristol, B52 8AE UK; 7 The National Institute for Health and Care Research Applied Research Collaboration West (NIHR ARC West) at University Hospitals Bristol and Weston NHS Foundation Trust, Bristol, UK; 8 Population Health Sciences, Bristol Medical School, University of Bristol, Bristol, BS1 1TH, B52 8EA UK; 9 Institute of Immunology and Immunotherapy, University of Birmingham, Birmingham, B15 2TT UK; 10 Queen Elizabeth Hospital, Birmingham, B15 2GW UK; 11 Institute of Metabolism and Systems Research, University of Birmingham, Birmingham, B15 2TT UK

**Keywords:** Self-management support, Diabetes, Underserved populations

## Abstract

**Aims:**

With numerous and continuing attempts at adapting diabetes self-management support programmes to better account for underserved populations, its important that the lessons being learned are understood and shared. The work we present here reviews the latest evidence and best practice in designing and embedding culturally and socially sensitive, self-management support programmes.

**Methods:**

We explored the literature with regard to four key design considerations of diabetes self-management support programmes: *Composition* - the design and content of written materials and digital tools and interfaces; *Structure* - the combination of individual and group sessions, their frequency, and the overall duration of programmes; *Facilitators* - the combination of individuals used to deliver the programme; and *Context* – the influence and mitigation of a range of individual, socio-cultural, and environmental factors.

**Results:**

We found useful and recent examples of design innovation within a variety of countries and models of health care delivery including Brazil, Mexico, Netherlands, Spain, United Kingdom, and United States of America. Within *Composition* we confirmed the importance of retaining best practice in creating readily understood written information and intuitive digital interfaces; *Structure* the need to offer group, individual, and remote learning options in programmes of flexible duration and frequency; *Facilitators* where the benefits of using culturally concordant peers and community-based providers were described; and finally in *Context* the need to integrate self-management support programmes within existing health systems, and tailor their various constituent elements according to the language, resources, and beliefs of individuals and their communities.

**Conclusions:**

A number of design principles across the four design considerations were identified that together offer a promising means of creating the next generation of self-management support programme more readily accessible for underserved communities. Ultimately, we recommend that the precise configuration should be co-produced by all relevant service and patient stakeholders and its delivery embedded in local health systems.

**Supplementary Information:**

The online version contains supplementary material available at 10.1186/s12939-023-01976-6.

## Introduction

In England the life expectancy of those with diabetes is improving amongst all age groups, including the circa 40,000 children and young people with diabetes (CYPD) [1]. However, the prognosis remains considerably worse for individuals from communities that are underserved by health services i.e., those who are economically deprived and/or from ethnic minorities that are engaged less effectively by formal healthcare interventions [2, 3], where they tend to have chronically higher glucose levels, and an increased risk of complications and death [4–8]. One way that the disparities in outcomes might be addressed, is by more effective utilisation of diabetes self-management support programmes (dSSP). Such multi-dimensional programmes which can equip patients with the confidence and ability to better manage both their symptoms and the psychological impact of their condition have demonstrated the potential to improve a range of clinical and behavioural outcomes across multiple chronic conditions including diabetes [9–12].

**Fig. 1:**
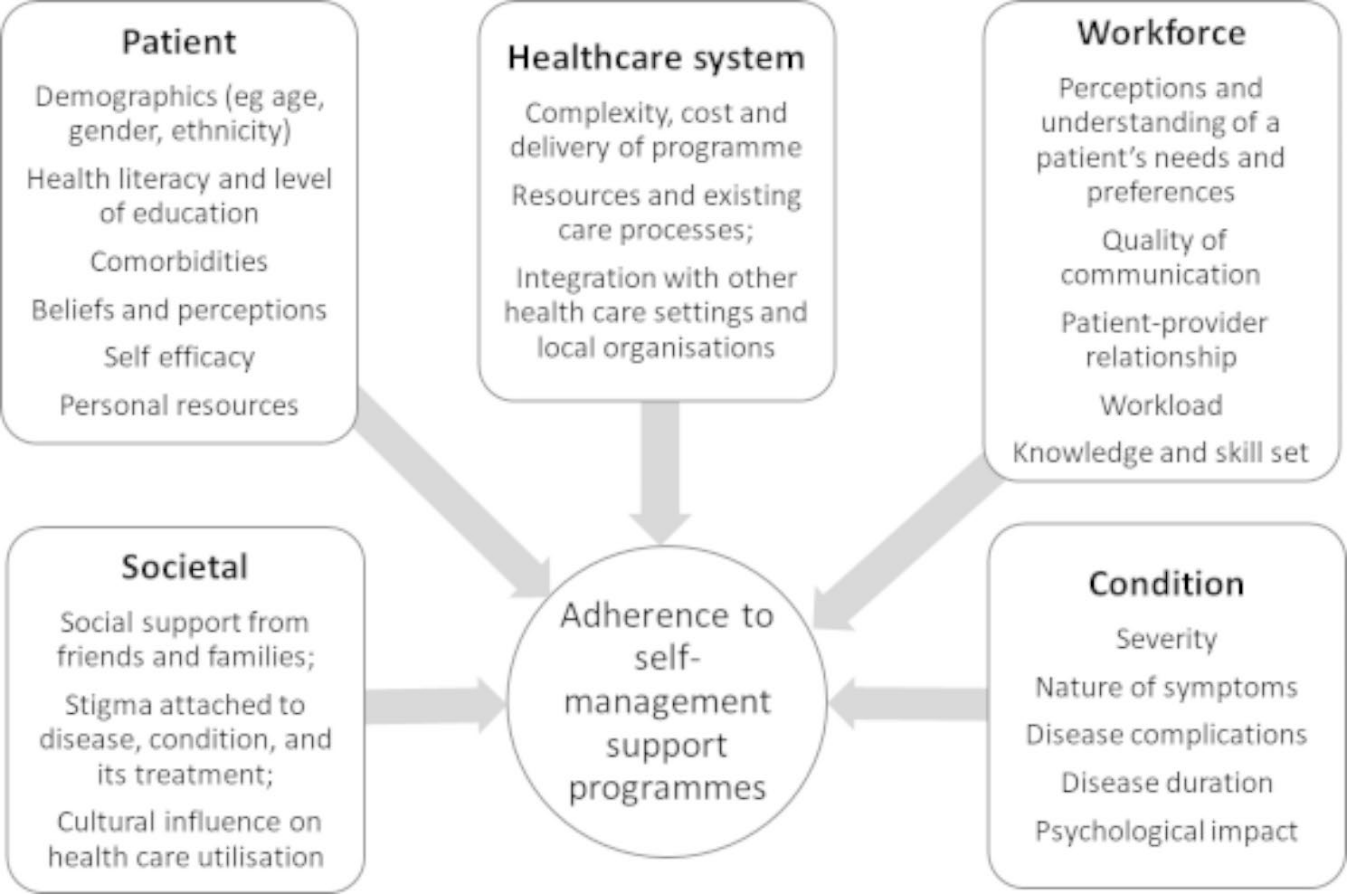
Contextual factors affecting access and engagement with (diabetes) self-management programmes (after [36–39])

In the United Kingdom (UK) there are a number of formal, nationally available dSSP [13–17] aimed at improving self-management across the whole population [18–20]. However, a number of contributory factors have been identified that influence consistent access, engagement, and ultimately adherence to these programmes, relating to the individual patient, the complexity of their condition, and the local health economy (see Fig. 1). For individuals with diabetes from underserved groups these barriers are exacerbated by the impact of a range of socio-economic, cultural and logistical issues that need to be addressed if the potential benefits of dSSP are to be realised and existing disparities mitigated [21–26]. Not all of these barriers can be overcome by a single dSSP, although it is now understood that more can be done in the design and delivery of self-management support to account for these challenges. Recent attempts have been made to adapt dSSP to better account for the cultural, environmental and social factors relevant to local populations [16, 27–29]. This has included using the inputs from target populations to develop programmes that better reflect the values, beliefs, and practices of local communities [30–33].

In attempting to design dSSP that better serve CYPD from underserved communities, the Diversity in Diabetes study is using the principles of engagement and co-design to create a bespoke programme of support, more sensitive to the needs and preferences of CYPD in the target populations (i.e. those from the two most deprived quintiles defined by the Index of Multiple Deprivation or from ethnically minoritized groups) and their families [34, 35]. To inform the co-production process, it is important to establish the latest evidence in designing culturally and socially sensitive dSSP. This narrative review provides a concise yet comprehensive summary of current knowledge and best practice in the composition, structure, and delivery of dSSP, and of the contextual factors that need to be accommodated in their design and implementation. It concludes by reflecting on the implications for creating and sustaining dSSP that are practical and appropriate for underserved communities in the UK.

## Methods

### Study design

The work consists of an integrative review of research conducted in populations with diabetes to determine the knowledge and ideas established in the design and implementation of dSSP for individuals from underserved communities [40, 41]. Our intention was not to identify every piece of work that has been conducted around dSSP for underserved populations, but to follow best practice in conducting integrative evidence reviews, summarizing the empirical and theoretical literature illustrated by recent and relevant examples to map the design principles currently being utilised within four key domains: These were informed by the existing self-management literature [9–12] and were selected and defined by the authors to enable an original and holistic description of the factors contributing to the design of a dSSP, which consisted of: *(1) Composition* of the written and digital materials including sentence structure and format and the use of images and graphics; *(2) Structure*, describing various elements in how the programme is delivered for example the number of individual or groups sessions, the location and duration of the SSP; *(3) Facilitators* referring to the identity and role of those delivering the SSP; and *(4) Context* which describes how the design of SSP accommodates social, cultural and health system influences. These are further described in Table 1.

**Table 1:** Design considerations for self-management support programmes for underserved populations

Domain	Definition	*Construct*	Definition
**Composition**	The principles employed in designing written materials and digital interfaces to maximise navigation, comprehension and assimilation.	*Syntactic structure and presentation of text*	The way sentences are constructed, and the vocabulary used. The choice of font, white space, and images.
		*Graphical-user interface*	The interactive display that enables a user to engage with electronic systems.
**Structure**	The combination of individual and group sessions, their duration and frequency, and the combination of taught elements	*Duration and location*	The length of time a course runs for, the number and length of individual sessions and their location including online.
		*Group or individual sessions*	The identity and numbers of those attending a taught component.
		*Syllabus*	The planned elements and aim(s) of the instruction including generic advice on living with long-term conditions, and specific skills relating to symptom management.
**Facilitators**	The combination of individuals used to deliver the programme	*Healthcare professionals*	Equipping health professionals that provide clinical care, with the ability to deliver self-management support.
		*Peer support*	Support from an individual who shares similar characteristics or experiences as a patient and/or a shared cultural and social background.
		*Community-based health workers*	These include local health service affiliated organisations such as pharmacists and voluntary services, community groups, and health workers.
**Context**	The impact of a range of individual and environmental factors on the successful delivery of SSP and sustained improvement in self-management practices	*Individual*	The clinical, psychosocial, and demographic characteristics that shape an individual’s response to their condition.
		*Community*	The characteristics of the local social, cultural, and built environment.
		*National and local health systems and economies*	The nature and quality of health care services, including the resources available, and their integration across settings and communities.

Where available, we report their impact on key diabetes related outcomes and consider the overall implications for the design of the next generation of SSP. Study eligibility criteria were established using the Population, Intervention, Comparison, Outcome, and Study design (PICO) framework [41] (see Table 2) and we have described our search in accordance with the Preferred Reporting Items for Systematic Reviews and Meta-Analyses (PRISMA) checklist [42].

**Table 2:** Summary of study eligibility

Type of study	Population or Problem	Intervention or Exposure	Comparison	Outcome
Systematic reviews including systematic reviews of reviews and systematic scoping reviews or primary research drawing on a range of methodologies including but not limited to RCTs, qualitative studies, and mixed methods.	Access, adherence, and engagement with SSP amongst individuals from underserved communities with diabetes or with other long-term conditions.	Elements of SSP developed or adapted to improve access, adherence and/or engagement and completion in underserved communities in four key domains relating to: Composition, Structure, Delivery, and Context	Routine care (including unsupported self-management) and/or routinely delivered self-management support programmes.	A range of self-management behaviours, psychological outcomes, and glycaemic control.

**Fig. 2:**
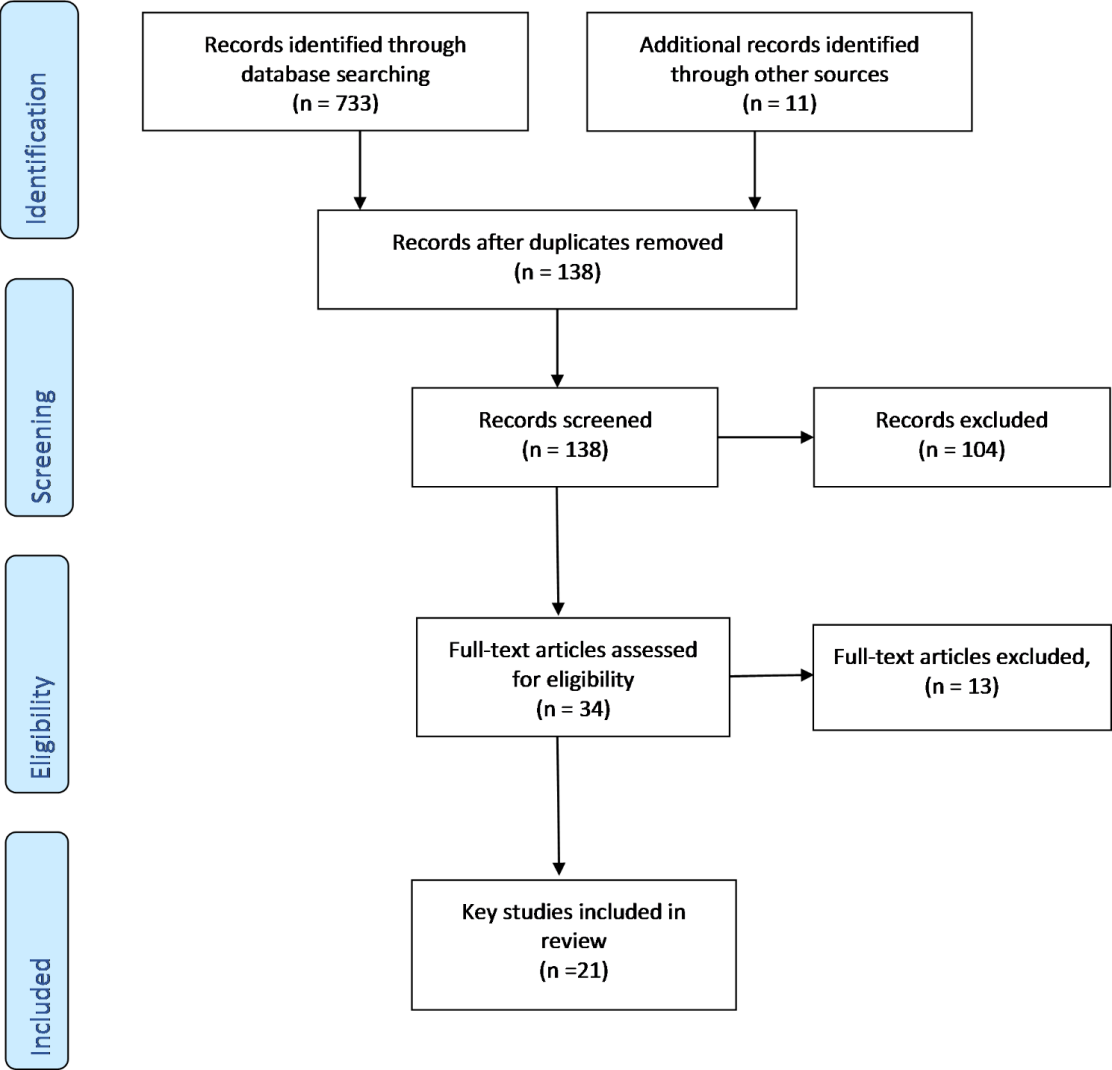
PRISMA diagram

### Search methods

The literature was searched in December 2022 from 2017 onwards for recent examples on the adaptations to dSSP related to the four considerations of design for underserved populations. This timespan allows us to describe recent research relevant to current models of healthcare delivery. We created a search for one database and adapted it for use in the others. used the following electronic medical databases: The Cochrane Library, MEDLINE, PubMed, CINAHL and EPPI. The inclusion criteria for our review comprised both primary research and a range of systematic reviews, that were peer-reviewed and published in English. The search terms can be found in Supplementary File 1.

### Data extraction and synthesis

The data was extracted within the four key design considerations by two authors (IL and SG). First, titles and abstracts were screened independently by IL and SG. The full text were then screened by IL with a second checking. A primarily narrative approach consistent with the recommended analytical method for narrative synthesis was used to summarise the nature and effect of the design elements within the four domains [40]. The criteria for selecting the data we reported were based on their relevance to the design and delivery of future programmes for underserved communities. We extracted data that included (i)programme overview (ii) author and publication date (iii) type of intervention (iv) target population (patient characteristic/condition, i.e., Type 1 or Type 2 Diabetes Mellitus) (v) quality score (vi) summary of effect.

## Results

A total of 21 papers describing the dSSP in underserved populations were included in the review. We initially retrieved 744 articles and after duplicates, protocols, or excluding because they were not specific to one of the design considerations or underserved populations were left with 21 examples explored in the review. The PRISMA Flow Diagram is shown in Fig. 2.

Below we describe the work conducted in designing dSSP to improve access, engagement, and adherence in underserved populations and discuss these adaptations in the context of their theoretical basis and what we know of dSSP in the general population. An overview of our findings is contained in Table 3, placing the adaptations in dSSP for underserved populations in the context of the potential barriers they are intended to address. There were 13 original papers and 7 reviews and referenced work conducted in Brazil, Mexico, Netherlands, Norway, Spain, United Kingdom, and United States of America (USA). Only 1 of the papers identified focussed on children 1. The characteristics of both the key reviews and primary research we included are summarised in Supplementary File 2.

**Table 3:** Summary of barriers to accessing dSSP and design related solutions

Barriers to dSSP access and engagement	Design consideration	Potential solution in designing dSSP for underserved populations
Health literacy, digital literacy, English as a second language, cognitive impairment	*Composition*: Syntactic structure	Use of tools designed to improve readability and navigation for those with lower (health) literacy [43, 44]. Use input of target diabetes population in the creation of written materials [45, 46] and incorporate their preferences and use the perspectives of patients from the target populations [33]
	*Composition*: Graphic User Interface	Use tools designed to improve usability of electronic interfaces [47, 48]. Appropriate combination of graphics, icons, and written elements and for diabetes patients in underserved populations quick access to information on glycaemic control, physical activity [49]
Inaccessible locations, unsuitable times	*Structure*: Duration and location	The use of community-based locations and a range of times [50–52]. mHealth can improve access [53–55] but with preferences for the use of portable technology [56] that can still be used offline [55]. Flexible programmes running for a number of sessions and varying in frequency of contact with facilitators have proven successful [57–[60]
The conflict between the benefits of shared experience of group sessions and the reluctance to identify with diabetes.	*Structure*: Group or individual sessions	Flexibility to meet preferences for individual or group sessions [61–[63]
The design and delivery of individual elements and/or the complexity of SSPs containing multiple elements	*Structure*: Syllabus	Preferences for content more relevant to their everyday lives [49, 64]. Creating SSPs with no more than three instructional elements [65].
The lack of awareness of health care professionals as to the importance of self-management	*Facilitator*: Health care professional	Use clearer marketing strategies, more effective referral pathways, and closer collaborations with clinicians [66].
Lack of understanding of personal circumstance	*Facilitator*: Peer supporters	Evidence of benefits for lifestyle behaviours [67, 68], reassurance [69] and accessing a range of underserved communities [70–73].
Lack of integration with community resources and local settings	*Facilitator*: Community-based health workers	Benefits of using CHWs included increased physical activity, improved dietary behaviours, glycaemic control [55, 58, 60, 74, 75] including amongst the elderly [76]. Also reported were the benefits of using community pharmacies [77]
Multiple demographic and socio-cultural influences on health engagement	*Context*: Individual	Use a range of validated tools to discern patient experiences and preferences [78–80]. Advantages of individual tailoring of education packages [81] and facilitators to patient preferences [82].
	*Context*: Socio-cultural	Advantages reported of tailoring SSP to reflect cultural needs and preferences at the population level [83–85]. A greater reliance on community educators, one-on-one interventions, visual information, alternative languages, and social support [86, 87].
The necessary resources, training, and processes specific to embedding SSP in health economies	*Context*: Health system	Increasing awareness of importance of SSP and maintenance of self-management skills amongst clinicians [88–91] and how socio-cultural influences impact self-management behaviours [85]. Realign targets to address the challenges reaching underserved populations, [92] build relationships with local groups at senior level [93].

### Composition

DSSP are reliant on a range of written content often presented alongside graphics, images and icons. These can be presented within printed materials such as booklets or handbooks, or digitally as part of a website, portal, or app.

#### Written materials

An individual’s ability to understand written content is linked to both the conceptual difficulty of the information and the cognitive demands of the chosen language, and the design, and format of its presentation [94]. Because reducing the cognitive load increases readability and engagement with health materials for all sections of the population, some generic rules can be applied to the syntactic content and structure. These include the use of shorter sentences and words, the avoidance of abbreviations and technical jargon, applying informal or conversational writing styles, and the use of patient stories in lieu of clinical facts and statistics [95–97].

Alongside linguistic considerations, elements of the presentation can also be adapted to assist comprehension [98]. For example; logically ordering content to help readers navigate the material [99]; surrounding text with white space, and using clear font and regular sub-headings to group text [44]. The judicious use of images can also increase understanding [100] though abstract graphics and symbols should be avoided in preference for simple line drawings which are closely linked to the relevant text and communicate a single idea [96, 101, 102].

#### Designing for underserved populations

Although the generic design principles outlined above should be routinely applied to all written materials they are particularly important where intended for underserved populations that characteristically exhibit lower health literacy, linked to their educational attainment, gender, levels of unemployment, and affiliation with religious beliefs [103][29]. There are several tools available to assess and improve the readability of written health materials such as the Flesch formula that uses the length of sentences and words to calculate the required reading level [43] or User-Testing where time to locate information by a sample of the target patient population is assessed [44]. A combination of these principles has been used recently in the creation of diabetes educational materials for CYPD in low income populations in South America [45].

#### Graphical user interface

The growing use of mHealth in dSSP (i.e., the use of apps, devices and digital connectivity to support healthcare) means that individuals with various information requirements, cognitive capabilities and limitations are increasingly likely to use graphical user interfaces (GUI) [104–106].The design principles used to increase comprehension and engagement with software based graphical interfaces are similarly informed by cognitive science [107]. They include the use of contrast between screen elements, grouping items, and using colours and graphics effectively and simply [108]. Previously the GUI of self-management tools for diabetes have improved engagement when they were combined with electronic reminders, tailored to individuals, and with clearer data visualizations and better organised text [109–111].

#### Designing for underserved populations

A number of tools that have been developed to support the process of designing GUI for users with low digital literacy, as found in underserved populations [2], these include design checklists [47], and tools that accurately assess the digital literacy of intended users [48]. In designing GUIs for underserved populations with diabetes, evidence from a recent engagement exercise in the USA described their preferences for interfaces that favour multimedia over text, and provide quick access to pertinent information on regulating blood glucose, diet, and physical activity [49].

### Structure

Typically, dSSP consists of a number of linked sessions delivered in various combinations of in-person and remote sessions over a period of weeks or months [16, 17].

#### Duration/location

The precise arrangement and combination of these taught and independently completed modules varies between programmes, with little consensus on the optimal duration and curriculum for maximising completion [112]. It is recognised that accessing dSSP via in-person sessions at central locations at fixed times, raises logistical barriers to access around transportation, inflexible work hours, and family commitments [113]. The flexibility offered by remote access to dSSP via a range of digital tools, text messaging, and telephone coaching [114–116] offers a promising means of improving access for all sections of the population [117, 118].

#### Designing for underserved populations

There is contradictory evidence around the optimum intensity of dSSP for underserved populations with successful programmes ranging from a minimum of ten in-person sessions delivered over six months to those running for shorter periods with varying frequencies of contact with facilitators [57–60, 119]. As the challenges of access to in-person elements of dSSP are more pronounced in underserved populations with reduced incomes and a greater reliance on public transport [61, 120], it is recommended they are offered sessions at different times and more readily accessible community facilities [50, 51]. There is growing evidence of the ability of mHealth to reach underserved populations with diabetes, with a number of systematic reviews of international evidence reporting improvements in diabetes control, healthcare utilization, and healthcare costs for Type 2 Diabetes Mellitus (T2DM) [53], younger patients with Type 1 Diabetes Mellitus (T1DM) [55] and older patients with T2DM [76], and hard to reach populations with T1DM and T2DM [121]. A systematic review of evidence in the USA that focussed on black and Hispanic patients reported similar positive outcomes [54] though a systematic review of web-based dSSP found that benefits were less evident in those groups of lower education or income [122].

Recent primary research exploring the preferences for mHealth and dSSP in underserved patient populations with T1DM, have expressed preferences for programmes that involved peers and family in support of their digital literacy [123], and young adults within the USA described the importance of improving the usability of mHealth technology to accommodate inconsistent internet connectivity [55], again in the USA Hispanic patients with T2DM described the need for lighter more portable technology [56].

#### Group vs. individual

The taught components of dSSP sessions can consist of group or one-to-one sessions, though the bulk of the evidence has described the benefit of structured group education which can improve a number of health status measures including psychological resilience, diet, physical activity, and symptom management [124, 125] and is more cost effective to deliver [126].

However, participation in group-based dSSP declines with lower economic status, advanced age, or if from an immigrant background [90, 127]. This may in part be due to the social comparisons inevitable in group work, which do not fit well with those not wanting to identify with having the condition [62, 128].

#### Designing for underserved populations

A recent systematic review of group sessions in dSSP described how they helped underserved populations with T2DM and significantly improved reductions in HbA1c by facilitating discussions and encouraging support from others facing the same socio-cultural challenges [63]. However, group participation can be stigma inducing for some sections of underserved populations and it is important to preserve the option of individual sessions [62]. (The cultural source of this stigmatisation is described in more detail in 4. Context)

#### Syllabus

DSSP typically incorporate several interacting components that address various requirements of self-management including diabetes education [129], self-monitoring [130], lifestyle changes [131, 132]; and psychological resilience [62]. However, the degree to which patients with diabetes engage with these multiple elements is influenced by a number of factors including education, self-efficacy, and level of (health) literacy and attention must be paid as to how these elements are combined [133].

#### Designing for underserved populations

As described previously, underserved populations tend to have lower levels of education and literacy skills than other sections of the population which adversely affects their ability to engage with complex programmes[49]. Attempts at exploring their preferences for how dSSP is structured, and the aspects of self-management important to them, have expressed preferences for practical and meaningful content, for example that which helps them maintain their independence or can directly inform healthy lifestyle choices [49, 64]. Studies investigating improving adherence to self-management programmes in a range of chronic conditions in underserved populations including diabetes, found that adherence can be improved by reducing the complexity of the programme, with evidence that the most effective programmes support only three or four self-management skills [65].

### Facilitators

DSSP can be delivered and supported by a combination of clinicians, peers and community-based health workers and pharmacists. In all cases, it is important the chosen facilitators are accessible, credible, and empathic with the group they are educating [134].

#### Healthcare professionals

Clinically trained health care professionals (HCP) are situated within the health service and can be directly involved in delivering taught components of dSSP, as well as contributing indirectly by supporting and complementing the messages and self-management skills being taught on dSSP through their routine contact with patients. The regularity of this contact, particularly in primary care environments means HCPs are well-placed to support patients in their identification and adherence to relevant self-management goals, and link them with local community and social groups [112, 135–137].

#### Designing for underserved populations

The role of HCPs in delivering or supporting self-management support in primary care and community settings remains less effective, in underserved communities in the USA [138]. Recent work trying to address this issue in the UK introduced an intervention designed to increase clinician engagement with dSSP in primary care organisations [139], which made recommendations for clearer marketing strategies that involved more coherent messaging around the benefits of self-management, more effective referral pathways that involved the ability to directly access booking systems, and closer collaborations with clinicians from other settings [66].

#### Peer support

Peer supporters i.e., those with similar characteristics as the target population and experiential knowledge of a specific illness or condition [140] are drawn from the communities they serve and so usually better understand the languages, cultures and circumstances of those they support [141]. There is growing evidence that peer facilitators can increase engagement and retention to dSSP amongst all sections of the populations, sharing practical experiences and helping people develop the skills and motivation needed to manage their health in the context of their everyday life [142, 143]. Benefits in a number of self-management behaviours have been consistently described [144–146].

#### Designing for underserved populations

There is also evidence of the value of peer support in reaching underserved populations with diabetes, in particular that they can help address some of the broader social determinants of health such as reducing isolation or providing confidence to adopt positive lifestyle behaviours and emotional reassurance [67, 69, 147]. A recent systematic review of peer support in dSSP amongst migrants and ethnic minorities, reported improvements in a range of lifestyle behaviours [68] and a number of recent studies in underserved populations in the USA have reported improvements in a range of clinical measures and diabetes-related behaviours in African-Americans [71], the rural poor [72] in diverse urban populations [70], and in Mexico in Mayan populations [73].

#### Community based health workers

Community Health Workers (CHWs) drawn from local populations and supported by the health system (but not necessarily a part of its organization) typically undergo shorter training than their professional colleagues [148]. Pharmacists can also be categorised as community-based care providers. The social support, accountability, practical skill building, and accessibility of CHWs has long-been recognised as a valuable adjunct to dSSP across all sections of the population [149] and more recently, local pharmacist-led interventions have also proven effective in improving medication adherence in adult patients with T1DM and T2DM [150].

#### Designing for underserved populations

Primary research has described how the use of CHWs has led to improved enrolment and engagement with dSSP in underserved communities in the UK [60]. Recent studies in the USA have also described how their involvement has led to increased physical activity and improved dietary behaviours amongst adults with low income [74] and clinically significant improvement in blood glucose control in Latino [58], ethnic minority [75], low-income, ethnic minority [55], and elderly populations [76].

A number of ongoing studies are exploring the impact of combining CHW led dSSP with mHealth in the USA [55, 151, 152], with social prescribing in the UK [153], with health coaches in the USA [154], and in community led initiatives in Norway [155]. Though little work to date, has focussed specifically on using pharmacists in underserved populations there are early indications, from a study in the USA, that where they share a language with the local population (“language concordance”), they can improve glycaemic control in minority populations [77].

### Context

Contextual influences, facilitate and constrain dSSP interacting with, and modifying the various elements of the programme [156]. Here we describe its effect within three domains: the individual, their community, including its societal and cultural aspects, and the broader health system.

#### Individual

Attendance and engagement to dSSP is impacted by the influences of a number of individual patient characteristics including their demographics [91, 157], clinical status [19], psychological factors [158, 159] and family and social support [111]. Many of the adverse impacts of these characteristics on dSSP engagement are exacerbated in underserved populations where they are compounded by a lack of awareness or understanding of the benefits of dSSP, feelings of stigma and shame, and the irrelevance of the advice of standardised programmes to their daily lives [61].

#### Designing for underserved populations

To support individuals within underserved populations to engage and adhere to dSSP, it is recommended that it is tailored to reflect the self-management support a particular individual prefers and needs [136]. This requires gaining a structured understanding of the outcomes important to that patient with diabetes [160] with a range of tools available and successfully used in diabetes to capture patient activation [79], patient outcomes [78], and health education impact questionnaires [80] as well as talking to patients about their personal narratives and emotional touch points [161]. Recent systematic reviews have described the success of tailored education packages in Latino populations in the USA [81] and the positive impacts on a range of self-management behaviours of ethnically matching facilitators’ ethnicity and language to Americans of African descent [82].

#### Community

The characteristics of a specific individual overlap and interact with the socio-cultural influences of their community as they engage with dSSP. These include the social conditions relating to the economic, environmental, and political features of their setting [157]. They also include the cultural influences of language, belief systems, and attitudes to health, care, and western medicine [33, 61, 157, 162, 163]: their precise nature varying according to their geographical origin, and religion [164–169].

#### Designing for underserved populations

Similar to the way in which dSSP can be tailored to meet the needs of individuals, they can be adapted to reflect cultural needs and preferences at the population level [83]. These adaptations include using ethnically relevant patient stories and presenting health-related issues in the context of broader social and cultural values [170]. Recent reviews have described how sensitively conducted, cultural tailoring can improve understanding of diabetes education in groups with lower health literacy [84] and overcome conflict between cultural preferences and health professional guidance in south Asians with T2DM in the UK [85].

#### Health system

For dSSP to be successful for any sections of a given community, it is important that national and local leaders commission programmes that are not only meaningful to local people but also embedded within the broader health system [113]. This often requires systemic change including a recognised need for more effective referral pathways, and building stronger links with the voluntary and community sector [171], and informational continuity between organisations and settings [172].

#### Designing for underserved populations

The ongoing issues with referral to dSSP are more pronounced amongst underserved populations as noted in the USA [88], the UK [89], and Canada where it has been suggested that clinicians are reluctant to refer those that have previously struggled to maintain appropriate health behaviours [90, 91]. It has been recommended that pathways to dSSP must be developed to better accommodate underserved populations by improving these referral processes and establishing more robust collaborative networks across statutory, voluntary, and community sectors [29, 173, 174].

## Discussion

### General findings

This overview of how dSSP can be developed to better engage underserved populations, proposes informing their design in four key areas. Firstly, *Composition*: it is important to reduce the cognitive load of written information and digital interfaces, making use of existing tools and the input of the target population. Secondly, the *Structure* needs to provide both group and individual options in programmes that are flexible in their duration, intensity, and utilisation of online resources in meeting the logistical pressure of physical access. Thirdly, selecting *Facilitators* should maximise the widely acknowledged benefits of using peers and community-based care providers (and a growing role for pharmacists) that mirror the characteristics of the populations they are educating. Fourthly, shaping dSSP according to *Context*, the importance of tailoring interventions sympathetic to the language, resources and beliefs of underserved populations was described alongside the need for a more integrated, whole system approach to dSSP implementation. Below we discuss the practical implications of how these considerations can be effectively implemented in the optimal combination of dSSP required by specific populations and suggest some practical steps to support their being embedded and sustained by the broader health service.

### Strengths and limitations

To aid our exploration, we categorised design considerations into four key areas to support those constructing dSSPs. This did mean that literature where these were not accurately described or defined was excluded. We also acknowledge that none of these elements exist in isolation and in reality, a single programme would utilise various combinations of these principles in an attempt to engage with underserved populations. It is also important to note that the term “underserved populations” reflects a heterogenous group defined by socio-economic status and a range of cultural factors, and that the whole population face the same barriers in accessing and engaging with dSSP as underserved groups but in the latter, they are exacerbated by socio-economic stressors and conflict with cultural expectations and requirements. This means that focussing on understanding and applying these design principles to meet the needs of localised underserved populations will likely generate learning that will improve adherence that can be applied to more affluent and culturally homogenous populations.

The time frame (five years) and geographical boundaries of the evidence we presented reflect the changing care environment and the growing interest in reaching underserved populations. Although much of the work we describe was undertaken in the USA within an insurance model of healthcare delivery, it can be argued that many of the barriers relating to accessing underserved populations are similar to those in other nations. To overcome the potential limitations of taking a cross-sectional approach to surveying the field, we have taken care to place our findings and recommendations in the context of existing knowledge, fulfilling our aim of producing a concise and coherent review of current evidence when considering the design of dSSP for underserved populations.

### Implications for future practice

#### Co-production and personalisation

The review demonstrated the wide range of options available in designing dSSP and the need to tailor programmes for underserved populations to reflect personal preferences and specific socio-cultural contexts [86, 87]. In considering the range of elements and adaptations available when compiling such programmes, it is important they are co-designed by a representative selection of stakeholders.

Ultimately the compilation of the dSSP must be consensually agreed by multiple stakeholders including commissioners, facilitators and target populations to ensure they remain acceptable, appropriate, and logistically and economically feasible [35]. Using co-design allows equal opportunity for all involved to reflect on and consensually agree the most appropriate elements and design solutions for any given programme [93]. This has been successfully used in the individual elements of dSSP in a range of underserved populations [30], for example in the creation of educational materials in Brazil [45, 46], lifestyle interventions in ethnic minorities in Finland [175] and the USA [176], and the design of mHealth innovations [177].

To support the compilation of the various elements into a coherent and socio-culturally sensitive programme for underserved populations with diabetes, two frameworks have emerged. Firstly, Lagisetty et al. have developed a framework that assesses the overall effectiveness of culturally tailored interventions for reaching underserved populations with diabetes [178]. It does this by unpacking the adaptation of the dSSP in terms of four domains Facilitator, Language, Location, and Message (or content) [178]. Secondly, the “Six G” framework developed by Gumber and Gumber performs a similar function in structuring the design of SSP for Black, Asian and Minority Ethnic (BAME) groups in the UK, namely Gender, Generation, Geographic origin, Genes, God (religion) and Gaps in knowledge and economic resources, [165]. The Diversity in Diabetes study will be using co-production techniques and a structured, framework-based approach to design a novel dSSP for CYPD from underserved populations, informed by the design principles outlined above. However, its precise configurations and the elements it contains will be a function of the co-design process.

#### Health care provider factors

If health care providers are to be actively engaged in co-production, there is a need to change long-standing attitudes toward self-management in the clinical workforce where previous evidence suggests dSSP can still be viewed as a “last resort” following major glycaemic crises or when traditional clinical treatment fails [36, 179, 180]. In convincing care providers of the legitimacy of supporting self-management as a professional priority, it has been suggested that practical support and time is ring-fenced to help them adapt systems and processes to more formally accommodate self-management support [181, 182]. It is also increasingly understood that to support self-management in underserved populations with diabetes, it is particularly important that clinicians are equipped to negotiate local socio-cultural influences on self-management behaviours [85] and such cultural competence should be embedded as a key skill set in delivering diabetes care [183].

#### Service-level factors

All of these design adaptations and the resulting dSSP will only be effective where the wider elements of the local health care system actively support their implementation [184, 185]. However, the financial implications of committing to the redesign of care processes and realignment of professional roles to support dSSP are considerable. Although economic analyses have found that the cost of developing and delivering dSSP is at least in part, offset by a subsequent reduction in health service utilisation [75, 186, 187], (aided by the use of novel modes of delivery such as mHealth [188] or pharmacies [189]) evidence of these savings is weak [68]. Too few studies that explore dSSP include explicit intervention costs (we found only one that directly addressed the issue) and senior-decision makers and commissioners remain reluctant to commit funding and resources to the long-term rewards of supporting dSSP in underserved populations [68]. One way this hesitancy might be reduced is by better targeted incentives realigned to address the challenges posed by cultural beliefs and practices [92, 190]. It has also been suggested previously that commissioners would also benefit from more concerted efforts to improve cultural understanding of local populations by strengthening and formalising relationships with local groups, religious bodies, and community leaders [93].

## Conclusions

We have described how evidence-based design of a programme of support can be used to address the challenges faced by underserved populations. It is important that any nascent programme attempting to reach the underserved, should engage with target populations in the consensual identification of potential solutions and the design of more precisely localised dSSPs.

### Electronic supplementary material

Below is the link to the electronic supplementary material.

Supplementary Material 1

Supplementary Material 2

## Data Availability

Not applicable.

## List of Abbreviations

CYPD: Children and young people with diabetes
CHW: Community-based health workers
CINAHL: Cumulative Index to Nursing and Allied Health Literature
dSSP: Diabetes self-management support programmes
EPPI: Evidence for Policy and Practice Information and Co-ordinating Centre
GUI: Graphical user interfaces
HbA1c: Haemoglobin A1c
HCP: Health Care Professionals
PICO: Population, Intervention, Comparison, Outcome, and Study design
PRISMA: Preferred Reporting Items for Systematic Reviews and Meta-Analyses
T1DM: Type 1 Diabetes Mellitus
T2DM: Type 2 Diabetes Mellitus
UK: United Kingdom
USA: United States of America
