# Supplementary material for: Current evidence for designing self-management support for underserved populations: an integrative review using the example of diabetes
Source: Int J Equity Health. 2023 Sep 11;22:188. doi: 10.1186/s12939-023-01976-6 (PMC10496394; doi:10.1186/s12939-023-01976-6)
Supplement: Supplementary file 1 — Supplementary Material 1 [file 12939_2023_1976_MOESM1_ESM.docx]

**Sample of search terms**

*Underserved populations*

("disadvantage"[All Fields] OR "disadvantageous"[All Fields] OR "disadvantageously"[All Fields] OR "disadvantages"[All Fields] OR "disadvantaging"[All Fields] OR "vulnerable populations"[MeSH Terms] OR ("vulnerable"[All Fields] AND "populations"[All Fields]) OR "vulnerable populations"[All Fields] OR "disadvantaged"[All Fields] OR ("underserved"[All Fields] OR "underserviced"[All Fields] OR "underservicing"[All Fields]) OR (("economical"[All Fields] OR "economics"[MeSH Terms] OR "economics"[All Fields] OR "economic"[All Fields] OR "economically"[All Fields] OR "economics"[MeSH Subheading] OR "economization"[All Fields] OR "economize"[All Fields] OR "economized"[All Fields] OR "economizes"[All Fields] OR "economizing"[All Fields]) AND ("deprival"[All Fields] OR "deprivation"[All Fields] OR "deprivations"[All Fields] OR "deprive"[All Fields] OR "deprived"[All Fields] OR "deprives"[All Fields] OR "depriving"[All Fields])) OR ("low"[All Fields] AND ("social class"[MeSH Terms] OR ("social"[All Fields] AND "class"[All Fields]) OR "social class"[All Fields] OR ("socio"[All Fields] AND "economic"[All Fields] AND "status"[All Fields]) OR "socio economic status"[All Fields]))) AND ("ethnical"[All Fields] OR "ethnically"[All Fields] OR "ethnicities"[All Fields] OR "ethnicity"[MeSH Terms] OR "ethnicity"[All Fields] OR "ethnic"[All Fields] OR "ethnics"[All Fields] OR "ethnology"[MeSH Subheading] OR "ethnology"[All Fields] OR "ethnology"[MeSH Terms]))) AND (y_5[Filter])

*Diabetes*

(("diabete"[All Fields] OR "diabetes mellitus"[MeSH Terms] OR ("diabetes"[All Fields] AND "mellitus"[All Fields]) OR "diabetes mellitus"[All Fields] OR "diabetes"[All Fields] OR "diabetes insipidus"[MeSH Terms] OR ("diabetes"[All Fields] AND "insipidus"[All Fields]) OR "diabetes insipidus"[All Fields] OR "diabetic"[All Fields] OR "diabetics"[All Fields] OR "diabets"[All Fields])

*Self-management*

("self management"[MeSH Terms] OR "self management"[All Fields] OR ("self"[All Fields] AND "management"[All Fields]) OR "self management"[All Fields]) AND ("program"[All Fields] OR "program s"[All Fields] OR "programe"[All Fields] OR "programed"[All Fields] OR "programes"[All Fields] OR "programing"[All Fields] OR "programmability"[All Fields] OR "programmable"[All Fields] OR "programmably"[All Fields] OR "programme"[All Fields] OR "programme s"[All Fields] OR "programmed"[All Fields] OR "programmer"[All Fields] OR "programmer s"[All Fields] OR "programmers"[All Fields] OR "programmes"[All Fields] OR "programming"[All Fields] OR "programmings"[All Fields] OR "programs"[All Fields])

*Elements of delivery*

("peer"[All Fields] AND ("support"[All Fields] OR "support s"[All Fields] OR "supported"[All Fields] OR "supporter"[All Fields] OR "supporter s"[All Fields] OR "supporters"[All Fields] OR "supporting"[All Fields] OR "supportive"[All Fields] OR "supportiveness"[All Fields] OR "supports"[All Fields])))

"health personnel"[MeSH Terms] OR ("health"[All Fields] AND "personnel"[All Fields]) OR "health personnel"[All Fields] OR ("healthcare"[All Fields] AND "provider"[All Fields]) OR "healthcare provider"[All Fields]

"community health workers"[MeSH Terms] OR ("community"[All Fields] AND "health"[All Fields] AND "workers"[All Fields]) OR "community health workers"[All Fields]

"written"[All Fields] AND ("health"[MeSH Terms] OR "health"[All Fields] OR "health s"[All Fields] OR "healthful"[All Fields] OR "healthfulness"[All Fields] OR "healths"[All Fields]) AND ("material"[All Fields] OR "material s"[All Fields] OR "materials"[All Fields])

(("web-based"[All Fields] OR ("digital"[All Fields] OR "digitalisation"[All Fields] OR "digitalised"[All Fields] OR "digitalization"[All Fields] OR "digitalize"[All Fields] OR "digitalized"[All Fields] OR "digitalizer"[All Fields] OR "digitalizing"[All Fields] OR "digitally"[All Fields] OR "digitals"[All Fields] OR "digitization"[All Fields] OR "digitizations"[All Fields] OR "digitize"[All Fields] OR "digitized"[All Fields] OR "digitizer"[All Fields] OR "digitizers"[All Fields] OR "digitizes"[All Fields] OR "digitizing"[All Fields]) OR ("australas plant pathol"[Journal] OR "app"[All Fields]) OR ("mhealth s"[All Fields] OR "telemedicine"[MeSH Terms] OR "telemedicine"[All Fields] OR "mhealth"[All Fields])) AND "2017/10/11 00:00":"3000/01/01 05:00"[Date - Publication]
