# Supplementary material for: Current evidence for designing self-management support for underserved populations: an integrative review using the example of diabetes
Source: Int J Equity Health. 2023 Sep 11;22:188. doi: 10.1186/s12939-023-01976-6 (PMC10496394; doi:10.1186/s12939-023-01976-6)
Supplement: Supplementary file 2 — Supplementary Material 2 [file 12939_2023_1976_MOESM2_ESM.docx]

**Table X Characteristics of key studies since 2017 (n=20)**

| ***Domain*** | ***Design consideration*** | ***Authors*** | ***Programme overview***  ***Target population (country)***  ***Review/primary research (study design)*** | **Age group  Condition**  **(Number of participants)** | **Date of search**  **Condition**  **(Number of studies/ study design)** | ***Primary outcomes***  ***(Effect size)*** | ***Direction of effect/comments*** |
| --- | --- | --- | --- | --- | --- | --- | --- |
| **Composition** | Syntactic structure | Moura, D. d. J. M., Moura, N. d. S., Menezes, L. C. G. d., Barros, A. A., & Guedes, M. V. C. (2017) | Development of a booklet on insulin therapy  Children low-middle income (Brasil)  Primary research (Qualitative) | Children (8-11)  T1DM  (19) |  | NA | Used the blogs of 7 children and adolescents with T1DM to create content which was refined via interviews with participants. |
|  |  | Roquini, G. R., Avelar, N. R. N., Santos, T. R., Oliveira, M. R. A. d. C., Galindo Neto, N. M., Sousa, M. R. M. G. C. d., & Trevisan, D. D. (2021) | Construction and validation of an educational booklet to promote adherence to oral antidiabetics.  Adults low -middle income (Brasil)  Primary research (Qualitative) | Adults (18+)  T2DM  (25) |  | Content Validity Index (CVI) (Valid score CVI score => 0.80) | Final booklet had CVI of 0.92 |
|  | GUI interface | Bonet Olivencia, S., Rao, A. H., Smith, A., & Sasangohar, F. (2021) | Eliciting Requirements for a Diabetes Self-Management Application  Underserved populations (USA)  Primary research (Qualitative) | Adults (18+)  T1DM and T2DM  (97 patients, 11 care providers) |  | Preferences for GUI design | Quick access to guidance on regulating blood sugar, diet, and exercise and physical activity  glucose monitoring  graphical visualisation and multimedia  Healthcare providers and patients held similar preferences |
| **Structure** | Duration and location | Mayberry, L. S., Lyles, C. R., Oldenburg, B., Osborn, C. Y., Parks, M., & Peek, M. E. (2019) | mHealth interventions for manging HbA1c  Disadvantaged and vulnerable people (USA, Canada, South Korea, Bangladesh, Iraq)  Review (Narrative) |  | January 2011- April 2019  T2DM  21 studies  (14 RCTs, 6 non-experimental, 1 stepped wedge) | Control of HbA1c | Findings based on 17 studies with a control group. 7 found improvements no meta-analysis |
|  |  | Pekmezaris, R., Williams, M. S., Pascarelli, B., Finuf, K. D 2020 | Adapting home telemonitoring interventions  Underserved Hispanic/Latino patients (USA)  Primary research (Qualitative) | 23 advisory board members  12 patients |  | Improving engagement with telecare | Recommends greater cultural sensitivity, tablet screens with improved usability Changes suggested to RCT structures to maximize participation relating to consent and recruitment strategies |
|  |  | Raymond, J. K., Reid, M. W., Fox, S., Garcia, J. F et al 2020 | Adapting home telehealth group appointment model  Low SES, publicly insured, minority young adults (USA)  Primary research (qualitative) | Young adults  (18-22)  T1DM  Participants numbers not described |  | Development of place-based self-management intervention | New model addresses the needs of high-risk YA in Southern California, with the goal of increasing access to care, improving follow-up frequency, and strengthening patient and provider satisfaction |
|  |  | Anderson, A., O’Connell, S. S., Thomas, C., & Chimmanamada, R. (2022) | Telehealth interventions to improve HbA1c  Black and hispanic patients (USA)  Review (Systematic and Meta-Analysis) |  | Up to March 2021  T2DM  10 studies (all RCTs) | Reduction in HbA1c  − 0.465 ([CI: − 0.648 to − 0.282], *p* = 0.000) | Telehealth interventions were delivered by telephone calls, text messages, web-based portals, and virtual visits. They typically involved diabetes self-management education |
|  | Syllabus | Schaffler, J., Leung, K., Tremblay, S., Merdsoy, L., Belzile, E., Lambrou, A., & Lambert, S. D. (2018). | Effectiveness of self-management interventions  Individuals with low health literacy and/or low Income (USA)  Systematic Review (Descriptive) |  | 2003-2018  T1DM and T2DM  N=8 (RCTs) | Range of self-management outcomes | Effective interventions included problem-solving/ taking action and/or resource utilization.  Efficacy did not vary by duration, format, or mode of delivery  No significant impact on HbA1c |
| **Facilitator** | Health care professional | Kurani, S. S., Lampman, M. A., Funni, S. A., Giblon, R. E., Inselman, J. W., Shah, N. D., Allen, S., Rushlow, D., & McCoy, R. G. (2021) | Diabetes care quality in primary care.  Socioeconomically deprived (USA) Primary research (quantitative, cross-sectional) | Adult (18+)  4090  T1DM and T2DM |  | Quality of diabetes care | Quality adversely affected by location (deprivation and rurality). |
|  | Peer supporters | Kowitt, S. D., Ayala, G. X., Cherrington, A. L., Horton, L. A., Safford, M. M., Soto, S., Tang, T. S., & Fisher, E. B. (2017) | Examining non-directive and directive peer support (USA)  Ethnic minorities and rural populations (USA)  Primary research (quantitative, structural equation modeling) | Adult (18+)  T2DM  (n=314) |  | Depressive symptoms (including diabetes distress) | Nondirective support contributed to lower depressive symptoms Directive support led to an increase in depressive symptoms |
|  |  | Rawal, L., Sahle, B. W., Smith, B. J., Kanda, K., Owusu-Addo, E., & Renzaho, A. M. (2021) | Lifestyle interventions self- management  Migrants and ethnic minorities in industrialized countries (USA,UK, Spain, Netherlands)  Review (systematic and meta-analyses) |  | 2000 and 2019  T2DM  17 studies (16 RCT, 1 quasi-experimental) | Control of HbA1c | small but statistically significant reduction in HbA1c level (-0.18%; 95% CI -0.32% to -0.04%, p=0.031). Sub-group analysis showed peer-led interventions showed relatively better HbA1c improvement than CHW (not statistically significant (p=0.379). |
|  |  | Nelson, L. A., Greevy, R. A., Spieker, A., Wallston, K. A (2021) | Effects of tailored text messaging  Diverse adults (USA)  Primary research  (quantitative, RCT) | Adult (18+)  T2DM  N=506 |  | Control of HbA1c | HbA1c was not significantly improved at 15 mths (−0.21%; 95% CI −0.78%, 0.36%; *P* = 0.475). Non-significant benefits in medication adherence and diet through 12 months and self-efficacy through 6 months. |
|  |  | Castillo-Hernandez KG, Laviada-Molina H, Hernandez-Escalante VM, Molina-Segui F, Mena-Macossay L, Caballero AE. | Peer support + Diabetes Self-management education (DSME)  Mayan populations (Mexico)  Primary research (Quantitative, RCT) | Adult (18+)  T2DM  N=58 |  | Control of HbA1c | Intervention group no statistical improvement, in A1C compared with the conventional DSME group. (Intervention participants exhibited statistically significant improvement in diabetes-related quality of life at 8 months.) |
|  | Community Health Workers | Gray, K. E., Hoerster, K. D., Taylor, L., Krieger, J., & Nelson, K. M. (2021) | Physical activity and dietary behaviors in a community health worker (CHW) led self-management A  Adults with low incomes (USA)  Primary research (Quantitative, RCT) | Adult (18+)  T2DM  (n=135 intervention, n= 142 usual care) |  | A range of lifestyle factors | Intervention participants engaged in more physical activity and reported better dietary behaviors (general diet, frequency of skipping meals, and frequency of eating out) at 12-months, |
|  |  | Allen, J. O., Concha, J. B., Mejía Ruiz, M. J., Rapp, A (2020) | Engagement in diabetes self-education  Underserved populations (USA)  Primary research (Qualitative) | Adult (18+)  T2DM  (Patients n= 22, health coaches n=3) |  | Engagement (completion of course) | Peers and coaches were important for social support and accountability.  accessible information, practical skill building, also identified as critical for engagement |
|  |  | Ye, W., Kuo, S., Kieffer, E. C., Piatt, G., Sinco, B., Palmisano, G., Spencer, M. S., & Herman, W. H. (2021). | Cost-effectiveness of dSSP led by CHWs and peer leaders  Latino (USA)  Primary research (Health economics) | Adult (18+)  T2DM  N=222 |  | Incremental cost-effectiveness ratio (ICER) | Community Health Worker + Peer Leader DSME/dSSP intervention improved health and better value than enhanced usual care |
|  |  | Slater, A., Cantero, P. J., Alvarez, G., Cervantes, B. S., Bracho, A., & Billimek, J. (2022) | Effectiveness of a community-initiated peer led self-management education program.  Latino (USA)  Primary research (Qualitative) | Adult 18+  T2DM  (n=688) |  | Control of HbA1c | At 14-week follow-up, mean decrease in HbA1c (95% CI) −1.1 (−1.3 to −0.9; *P* < .001) in the intervention compared to control −0.3 (−0.4 to −0.2; *P* < .001) in the comparison cohort |
| **Context** | Individual | Hildebrand, J. A., Billimek, J., Lee, J.-A., Sorkin, D. H., Olshansky, E. F., Clancy, S. L., & Evangelista, L. S. (2020) | Effect of diabetes self-management education on glycemic control  Latino adults (USA)  Review (systematic + meta-analysis) |  | January 1997 and March 2019  T2DM  Adult 18+  N=23 studies (2 quasi-experimental, 3 pilot RCTs, 1 feasibility study, 17 RCTs). | Control of HbA1C | 18 studies included in in meta-analysis Pooled estimate effect of DSME on A1C from the random effect model was −0.240 (95% confidence interval = −0.345, −0.135, *p* < 0.001).  Subgroup analyses demonstrated greater A1C reductions in studies with intervention duration ≤6 months, |
|  |  | Wadi, N. M., Asantewa-Ampaduh, S., Rivas, C., & Goff, L. M. (2022). | Culturally tailored lifestyle interventions for prevention and management of diabtetes.  Adults of black african ancestry (USA)  Review (systematic review) |  | Up to October 2020  T2DM  Adult 18+  N=16 (RCTs) | Tailoring methods and their effectiveness | dSSP with ethnically matched facilitators and that tailored more than one element showed the greatest HbA1C benefits |
|  | Community | Patel, T., Umeh, K., Poole, H., Vaja, I., & Newson, L. (2021) | Impact of cultural identity conflict on engagement with self-management  South Asian (UK)  Review (Interpretative Synthesis) |  | Up to May 2020  T2DM  N=19 (qualitative) | Psychology underpinning T2D self-management behaviours | Patients “reconstruct knowledge” to manage their psychological, behavioural, and cultural conflicts with healthcare advice on Type 2 diabetes |
|  |  | Goff, L. M., Moore, A., Harding, S., & Rivas, C. (2020) | Culturally sensitive DSME  Black african and caribbean communities (UK)  Primary research (qualitative) | Health care providers  T2DM  n=10 |  | Experiences of delivering DSME to target population | The pressure of growing numbers of patient’s vs targets unsuited to diverse communities; distrust of conventional medicine; benefits of racial concordance and cultural knowledge; the need to address gaps in structured education. |
